# Automated computational analysis reveals structural changes in the enteric nervous system of nNOS deficient mice

**DOI:** 10.1038/s41598-021-96677-x

**Published:** 2021-08-25

**Authors:** Ben R. Cairns, Benjamin Jevans, Atchariya Chanpong, Dale Moulding, Conor J. McCann

**Affiliations:** Stem Cells and Regenerative Medicine, UCL Great Ormond Street Institute of Child Health, 30 Guilford Street, London, WC1N UK

**Keywords:** Machine learning, Network topology, Microscopy, Disease model, Enteric nervous system, Large intestine, Gastroenterology, Gastrointestinal models

## Abstract

Neuronal nitric oxide synthase (nNOS) neurons play a fundamental role in inhibitory neurotransmission, within the enteric nervous system (ENS), and in the establishment of gut motility patterns. Clinically, loss or disruption of nNOS neurons has been shown in a range of enteric neuropathies. However, the effects of nNOS loss on the composition and structure of the ENS remain poorly understood. The aim of this study was to assess the structural and transcriptional consequences of loss of nNOS neurons within the murine ENS. Expression analysis demonstrated compensatory transcriptional upregulation of pan neuronal and inhibitory neuronal subtype targets within the *Nos1*^*−/−*^ colon, compared to control C57BL/6J mice. Conventional confocal imaging; combined with novel machine learning approaches, and automated computational analysis, revealed increased interconnectivity within the *Nos1*^*−/−*^ ENS, compared to age-matched control mice, with increases in network density, neural projections and neuronal branching. These findings provide the first direct evidence of structural and molecular remodelling of the ENS, upon loss of nNOS signalling. Further, we demonstrate the utility of machine learning approaches, and automated computational image analysis, in revealing previously undetected; yet potentially clinically relevant, changes in ENS structure which could provide improved understanding of pathological mechanisms across a host of enteric neuropathies.

## Introduction

Nitrergic neurons, which express neuronal nitric oxide synthase (nNOS), play a predominant role in inhibitory neurotransmission within the enteric nervous system (ENS)^1–5^. Through the production and release of nitric oxide (NO), nNOS^+^ neurons relax smooth muscle and play a major role in establishing “normal” motility across the gastrointestinal (GI) tract^6–9^. Importantly, loss of nNOS neurons has been reported in a range of human enteric neuropathies which can affect any region of the gut^10^, including oesophageal achalasia^11^, gastroparesis^12^, slow transit constipation^13^, Chagistic megacolon^14^ and Hirschsprung disease^15,16^. However, it is well established that neurons within the ENS display distinct expression patterns, known as the “chemical code”, whereby individual neurons display colocalization of different neuronal markers. Despite the multiple reports of loss of nNOS neurons in disease, the effects of such loss on the composition, and indeed structure, of the ENS remain poorly understood. This is in part due to the inherent complexity of the ENS, in terms of its structural architecture and the plurichemical nature of its neurotransmitter expression pattern.

nNOS which is encoded by the *NOS1* gene has been shown to be highly conserved across vertebrate species^17^. Notably, *Nos1 (nNOS)* deficient mice have been shown to exhibit the clinical phenotype of a number of human gut motility disorders including delayed gastric emptying^8,18,19^, and slow transit in the colon^7,20^. The use of this global *Nos1*^*−/−*^ knockout model therefore provides an opportunity to examine differences in the ENS, after specific loss of nNOS signalling. In this study we combine conventional imaging, and molecular techniques, with machine learning analytical approaches; which allow in-depth, high-throughput, analysis of largescale imaging datasets, to demonstrate the consequences of global loss of nNOS expression in the ENS. We show that knockout of *Nos1* leads to compensatory transcriptional upregulation of neurotransmitter genes. Further, we describe occult structural changes within the ENS of *Nos1*^*−/−*^ mice. Thus, we propose that disruption of specific neuronal subtypes can have wide ranging; potentially clinically relevant, effects within the ENS which are often undetected.

## Results

### Gross characterisation of the ENS in the ***Nos1***^***−/−***^ colon

**Figure 1:**
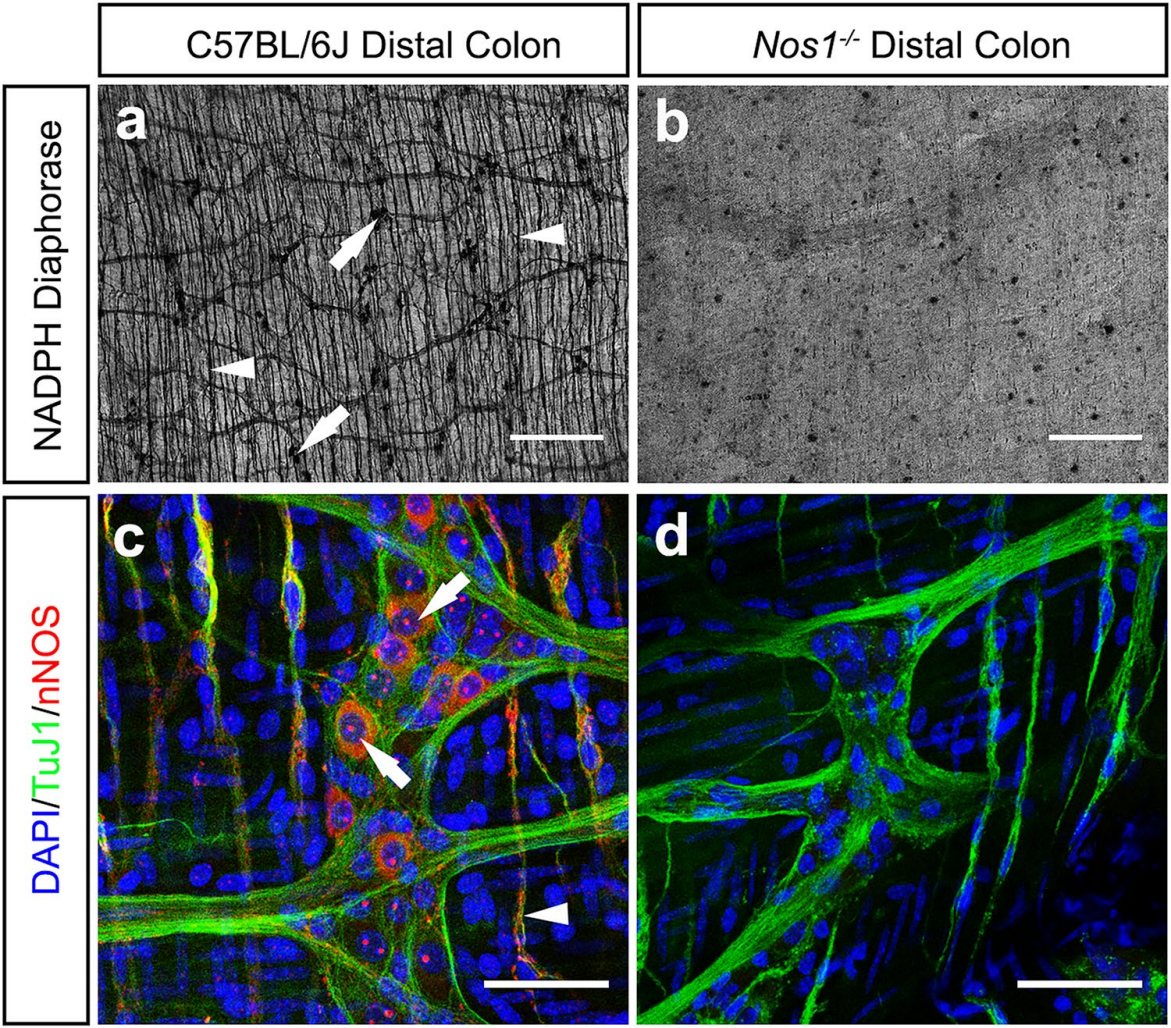
*Nos1*^*−/−*^ mice display complete loss of nNOS neurons in the distal colon. (**a**) Representative low-power image of NADPH diaphorase staining in wild-type C57BL/6J. nNOS^+^ cell bodies (arrows), within enteric ganglia, and nNOS^+^ fibres (arrowheads) are indicated. (**b**) Representative low-power image of *Nos1*^*−/−*^ distal colon demonstrating loss of nitrergic neurons as seen with the absence of NADPH diaphorase staining. (**c**) Representative high-power confocal z-stack image demonstrating the presence of nNOS^+^ (red) neuronal cell bodies (arrows) within TuJ1^+^ (green) ganglia structures at the level of the myenteric plexus. nNOS^+^TuJ1^+^ intramuscular nerve fibres could also be observed in the C57BL/6J colon (arrowhead). (**d**) Representative high-power confocal z-stack image demonstrating the absence of nNOS^+^ immunoreactivity within the *Nos1*^−/−^ colon. In the absence of nNOS^+^ both TuJ1^+^ ganglia-like structures and intramuscular neurons can be observed in *Nos1*^−/−^ colonic tissue. Blue = DAPI. Scale bars, 500 μm (**a**,**b**) 50 μm (**c**,**d**).

**Figure 2:**
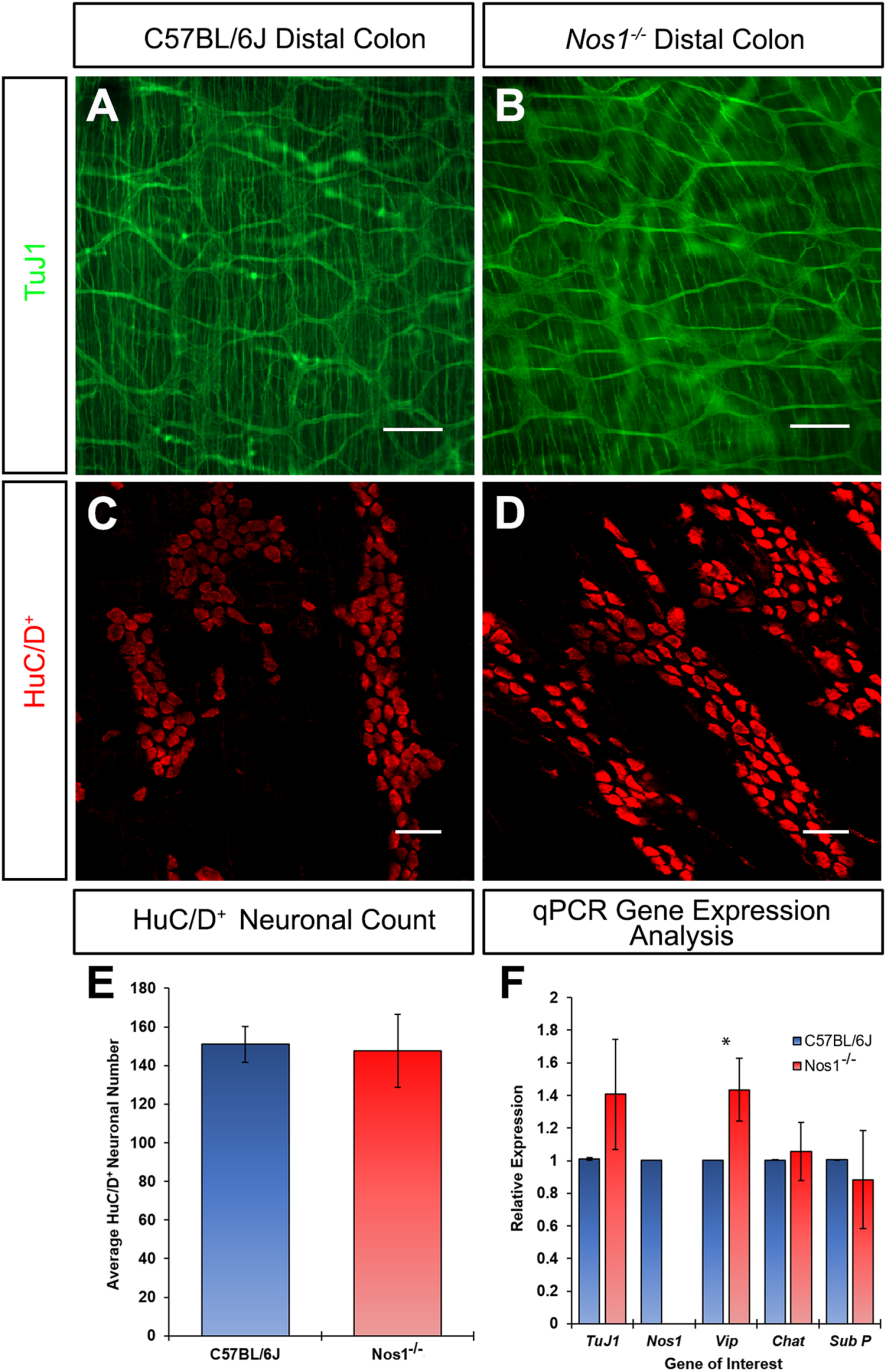
The ENS in *Nos1*^*−/−*^ distal colon is visually comparable to C57BL/6J mice but displays transcriptional compensation. (**a**,**b**) Representative montages of stitched low-power images showing the TuJ1^+^ (green) ENS in the C57BL/6J (*a*) and *Nos1*^*−/−*^ (*b*) distal colon. (**c**,**d**) Representative high-power confocal image showing HuC/D^+^ (red) neuronal cell bodies, at the level of the myenteric plexus, in the C57BL/6J (*c*) and *Nos1*^*−/−*^ (*d*) distal colon. (**e**) Summary data of HuC/D^+^ neuronal cell counts in the C57BL/6J (blue bar, 25hpf, n = 5) and *Nos1*^*−/−*^ (red bar, 25hpf, n = 5) distal colon. P > 0.05 by Student’s t-test. (**f**) Gene expression analysis in C57BL/6J (blue bars, n = 3) and *Nos1*^*−/−*^ (red bar, n = 3) distal colon showing mRNA fold change (ΔΔCT) of major excitatory and inhibitory neuronal subtypes. Error bars represent mean ± s.e.m. in all panels. ^*^P ≤ 0.05 comparing ΔCT values by Welch’s t-test. Scale bars, 200 μm (**a**,**b**) 50 μm (**c**,**d**).

Characteristically, the distal colon of C57BL/6J mice contains a dense network of nNOS^+^ cell bodies (Fig. 1a,c; arrows) within ganglionic structures, at the level of the myenteric plexus; and intramuscular fibres (arrowheads), as observed by NADPH diaphorase staining. By contrast, *Nos1* deficient mice (*Nos1*^*−/−*^) mice display complete loss of NADPH diaphorase activity and nNOS^+^ neurons in the distal colon (Fig. 1b,d)*.* Despite the complete loss of nNOS neurons in the *Nos1*^*−/−*^ colon the gross morphology of the enteric neuronal network remains visually comparable to that of C57BL/6J tissues as revealed by TuJ1 immunolabeling (Fig. 2a,b)*.* To determine potential differences in neuronal numbers we examined HuC/D^+^ neuronal cell counts. Interestingly, within the *Nos1*^*−/−*^ distal colon we observed similarly patterned ganglia structures (Fig. 2c,d) and comparable neuronal cell numbers (148 ± 19; 25 hpf, n = 5) when compared to the C57BL/6J distal colon (151 ± 9; 25 hpf, n = 5; P = 0.927; Fig. 2e).

### Molecular expression of major neuronal subtypes in *Nos1*^*−/−*^ colon

Having shown that neuronal cell numbers are comparable in the ENS of the distal colon, in both C57BL/6J and *Nos1*^*−/−*^ mice, we next sought to determine the transcriptional effects of loss of nNOS using qPCR. Despite being visually comparable in terms of neuronal network structure, *TuJ1* expression was found to be approximately 1.4-fold (1.41 ± 0.34, n = 3) greater in the *Nos1*^*−/−*^ distal colon compared to C57BL/6J tissue (1.01 ± 0.01, n = 3; P = 0.746; Fig. 2f). In further assessment of the major excitatory and inhibitory neuronal subtypes we observed that *ChAT* (1.06 ± 0.18, n = 3; P = 0.982) and *Sub P* (0.88 ± 0.30, n = 3; P = 0.481) were expressed at similar levels in the *Nos1*^*−/−*^ distal colon when compared to C57BL/6J tissue (1.00 ± 0.0 and 1.00 ± 0.0 respectively, n = 3; Fig. 2f). As expected, *Nos1* expression was not observed (0.00 ± 0.0, n = 3; Fig. 2f). However, in the absence of *Nos1* we observed a 1.43-fold increase in the relative expression of *Vip* (1.43 ± 0.19, n = 3; P = 0.045), compared to C57BL/6J tissues (1.00 ± 0.00, n = 3; Fig. 2f). These results combined, suggest that while overall gross network morphology remains similar, and neuronal numbers remain largely unchanged, subtle changes in network dynamics and compensatory mechanisms occur upon the loss of nNOS signalling in the *Nos1*^*−/−*^ distal colon.

### Machine learning analysis reveals occult changes in the *Nos1*^*−/−*^ ENS

**Figure 3:**
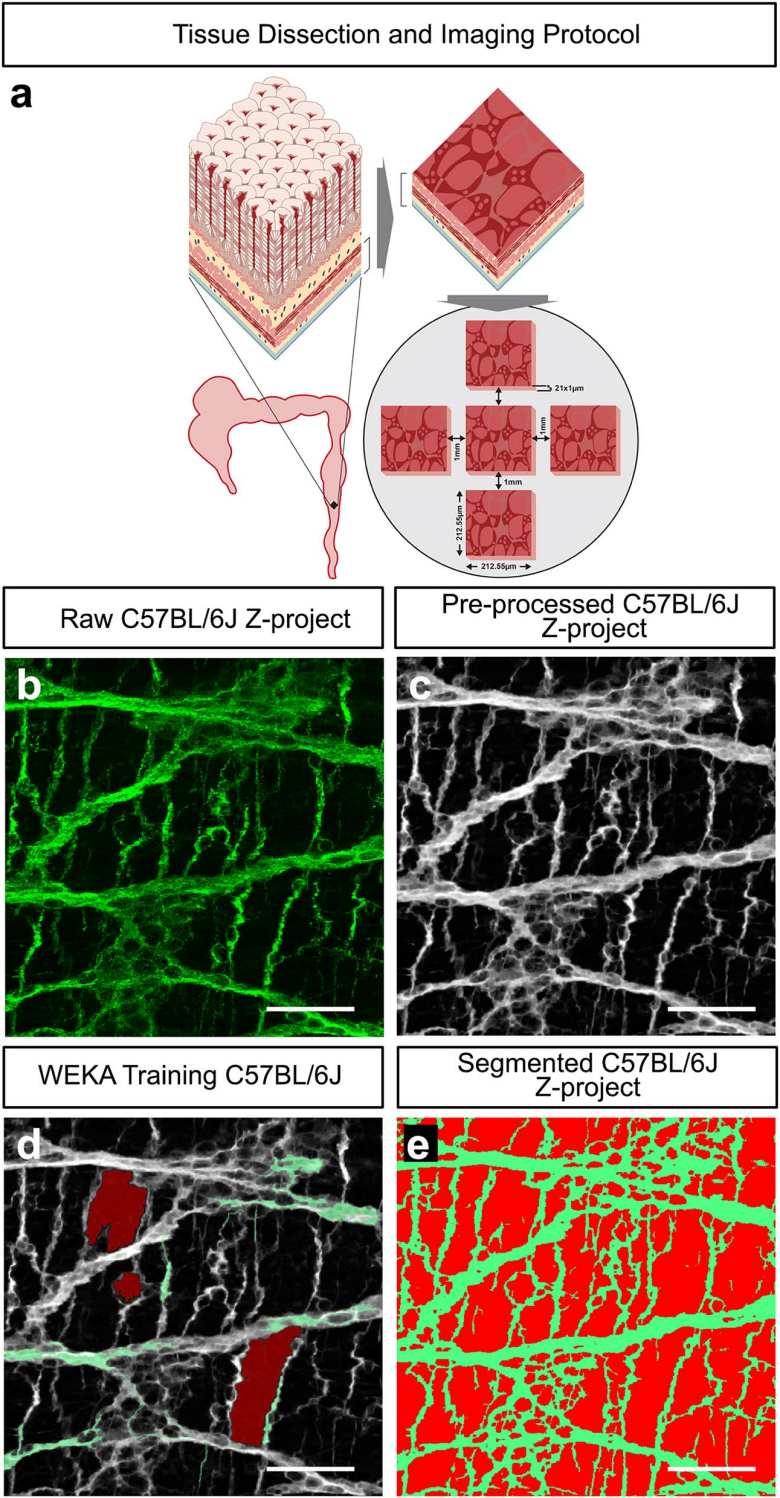
Imaging and segmentation protocol for high-throughput machine learning analysis of the ENS. (**a**) Schematic illustration demonstrating the isolation and dissection of the distal colon along with the confocal imaging protocol used to capture the ENS. Illustration created by UCL Medical Illustration. (**b**) Representative high-power confocal raw z-projection demonstrating the presence of the TuJ1^+^ (green) ENS in the C57BL/6J distal colon. (**c**) Representative pre-processed image, of *b,* following filtering to remove noise and improve segmentation. (**d**) Visual representation of WEKA Segmentation process showing segmentation training of the TuJ1^+^ network (green) and background (TuJ1^−^; red) from the filtered image shown in *c*. (**e**) Representative image of digitally segmented image *d* showing pseudocoloured TuJ1^+^ neural network (green) and background (red). Scale bars, 50 μm.

**Figure 4:**
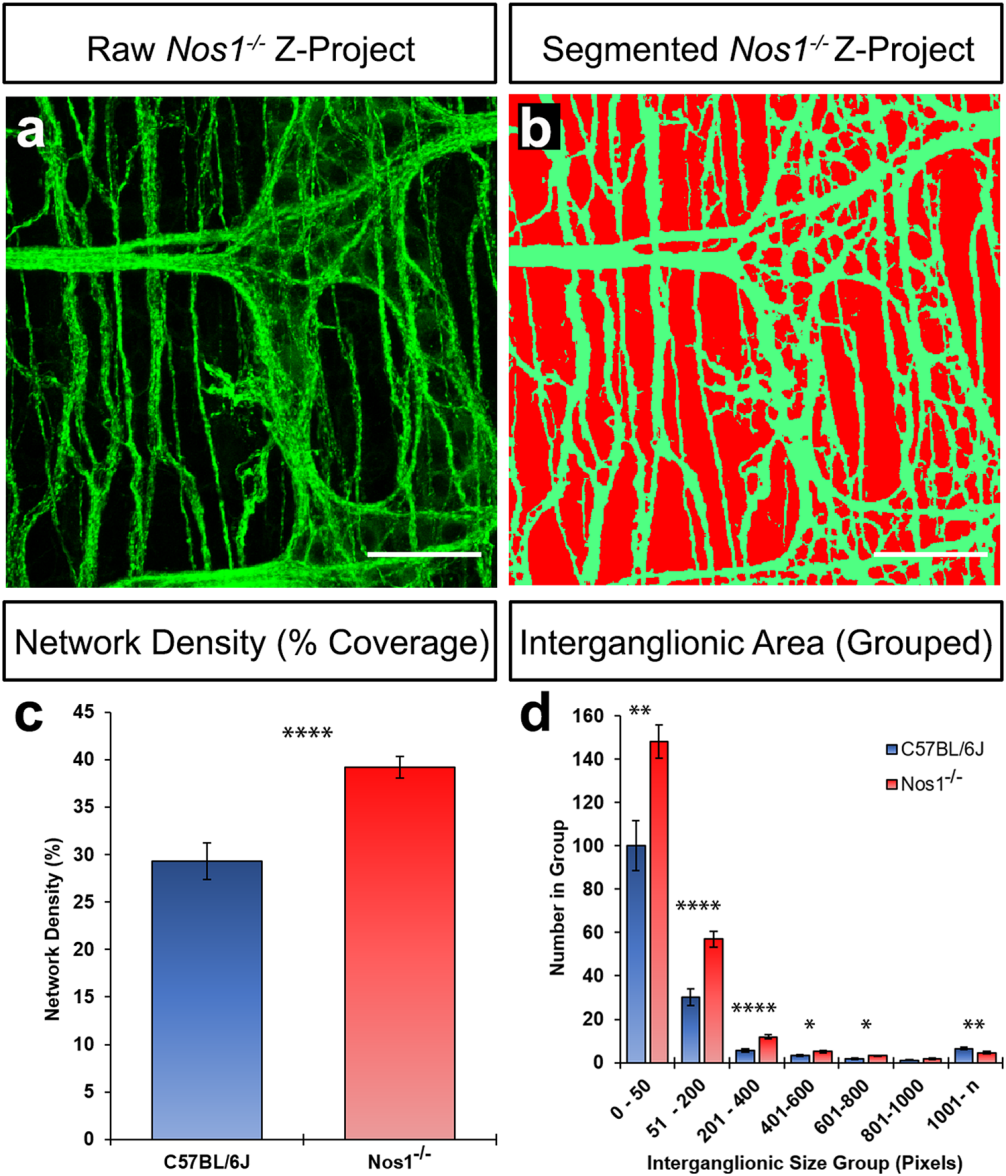
The *Nos1*^*−/−*^ distal colon displays increased ENS coverage. (**a**) Representative confocal z-project demonstrating the TuJ1^+^ (green) network in the *Nos1*^*−/−*^ distal colon. (**b**) Representative image of digitally segmented image in *a* showing pseudocoloured TuJ1^+^ neural network (green) and “interganglionic” regions (i.e., background; red). (**c**) Summary data showing network density (%) in C57BL/6J (blue bar) and *Nos1*^*−/−*^ (red bar) mice. (**d**) Summary data showing the number of binned interganglionic areas (pixel units; pxls) in C57BL/6J (blue bar) and *Nos1*^*−/−*^ (red bar) distal colon. Bins represent very small (0–50 pxls), small (51–200 and 201–400 pxls), medium (401–600 and 601–800) and large (801–1000 & 1000-n) interganglionic areas. *P ≤ 0.05, **P ≤ 0.01, ****P ≤ 0.0001 by Welch’s Student t-test Scale bars, 50 μm.

In order to assess if changes in ENS composition, in terms of neuronal subtype gene expression, leads to changes in ENS structure which are visually indistinguishable, we used readily available machine learning tools; combined with in-house scripting (Supplementary Information), to quantitatively compare ENS network characteristics (e.g., network density, interganglionic area, network branching, junctioning, directionality and coherence) in multiple data sets (i.e., 105 optical sections/distal colon) in both control C57BL/6J and *Nos1*^*−/−*^ distal colon. To ensure reproducibility and capture the 3D-structure of the distal colonic ENS, we devised an imaging protocol whereby 21 optical sections (1 μm) were obtained in a cross-shaped array (Fig. 3a) using confocal microscopy. Following acquisition, imaged stacks were analysed after pre-processing and segmentation (Fig. 3b–e). Interestingly, while visually comparable, machine learning digital analysis revealed that TuJ1^+^ network density is significantly greater in the *Nos1*^*−/−*^ distal colon (39.2 ± 1.1%; 30 z-projected hpf, n = 6) compared to C57BL/6J controls (29.3 ± 1.9%; 30 z-projected hpf, n = 6; P < 0.0001; Fig. 4a–c). To validate our machine learning segmentation and network coverage analysis, we analysed equivalent images taken from the colon (ganglionic, transitional zone and aganglionic regions) of an *Ednrb*^*−/−*^ (*Endrb*^*tm1Ywa*^*/*J) mouse model which displays variable but graded loss of the ENS along the length of the colon. Here, as expected, our digital analysis revealed that network density was reduced in a graded fashion when comparing ganglionic (18.6%), transition zone (16.7%) and aganglionic (10.1%) regions confirming the validity of our approach (Supplementary Figure 1).

Given the changes observed in TuJ1^+^ network coverage in the *Nos1*^*−/−*^ distal colon, we also analysed the negative space (i.e., TuJ1^−^ pixels) as a proxy for interganglionic area. Interganglionic areas were binned to seven groups according to their size (pixel number). Compositional changes in the interganglionic area were also found to be significantly different, with *Nos1*^*−/−*^ mice having more numerous, but smaller interganglionic areas; with significantly fewer large (> 1000pxls) areas (Fig. 4d). Notably, areas designated as “very small” (i.e., < 50 pixels) were found to be increased in the *Nos1*^*−/−*^ ENS (148.00 ± 7.92) when compared to the C57BL/6J mice (100.00 ± 11.75; 30 z-projected hpf, n = 6; P = 0.001; Fig. 4d). Similarly, “small” areas (i.e., 51–200 and 201–400-pixels) were also found to be more numerous (p =  < 0.0001) in *Nos1*^*−/−*^ distal colon with 56.90 ± 3.56 or 11.90 ± 0.90 per respective group compared to C57BL/6J distal colon (30.17 ± 3.93 and 5.53 ± 0.71 respectively; 30 z-projected hpf, n = 6; Fig. 4d). Medium sized areas (i.e., 401–600 and 601–800 pixels) were found to be increased in (p < 0.05) in *Nos1*^*−/−*^ mice with 5.07 ± 0.45 (P = 0.014) and 3.13 ± 0.31 (P = 0.016) per respective group vs 3.30 ± 0.51 and 1.90 ± 0.38 in C57BL/6J distal colon (30 z-projected hpf, n = 6; Fig. 4d). By contrast, interganglionic spaces with pixel sizes between 801–1000 were comparable between *Nos1*^*−/−*^ (1.77 ± 0.0.23) and C57BL/6J (1.17 ± 0.28; P = 0.108, 30 z-projected hpf, n = 6; Fig. 4d) mice. However, larger interganglionic areas (i.e., > 1000pxls) were found to be significantly reduced in the *Nos1*^*−/−*^ distal colon (4.60 ± 0.44) compared to C57BL/6J (6.50 ± 0.48; P = 0.006, 30 z-projected hpf, n = 6; Fig. 4d) mice.

### Loss of *Nos1* expression causes changes in ENS branching and junctions

To determine how the observed alterations in overall network coverage reflect specific changes in network composition, we further quantified branch number, branch length and the number of junctions present within the ENS.

**Figure 5:**
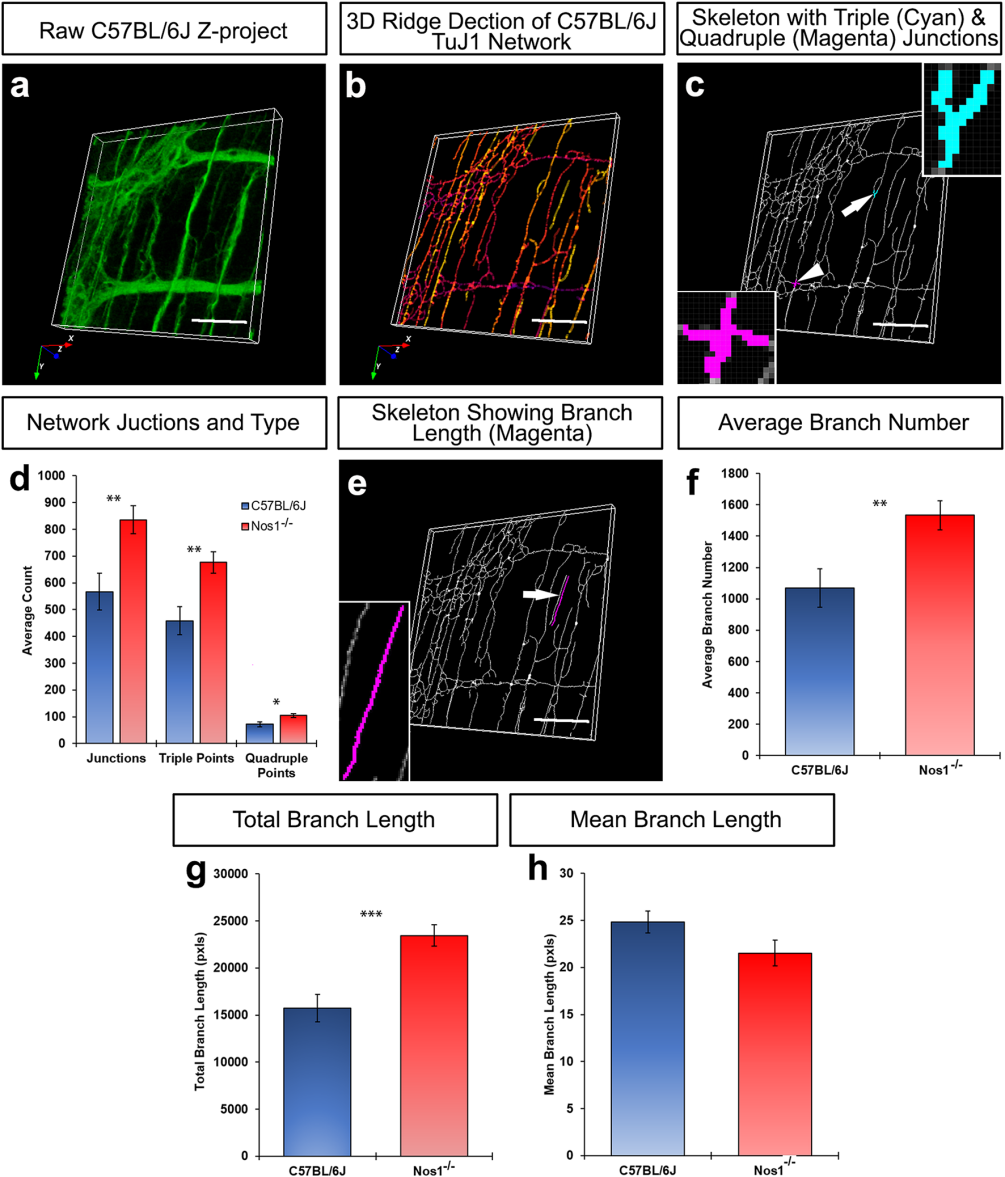
Altered ENS branching and junctioning in the *Nos1*^*−/−*^ distal colon. (**a**) Representative confocal 3D view demonstrating the TuJ1^+^ (green) network in the C57BL/6J distal colon. (**b**) A twice eroded skeletonised version of *a,* produced through ridge detection, projected in 3D and pseudocoloured in ‘mpl-plasma’. Colours represent z-depth with yellow being the most superficial structures, moving gradually to purple with increasing z-depth. (**c**) A binary, true skeletonised network (i.e., 1 pixel) of *a.* Upper inset shows a zoomed image of a representative pseudocoloured (cyan) triple junction (arrow). Lower inset shows a zoomed image of a representative pseudocoloured (magenta) quadruple junction (arrowhead). (**d**) Summary data showing junctional analysis between C57BL/6J (blue bars) and *Nos1*^*−/−*^ (red bars). (**e**) Representative image of binary, true skeletonised network of *a* showing a representative pseudocoloured (magenta) branch (arrow). (**f**–**h**) Summary data showing total branch number (*f*), total branch length (*g*) and mean branch length (*h*), in C57BL/6J (blue bars) and *Nos1*^*−/−*^ (red bars) mice. *P ≤ 0.05, **P ≤ 0.01, ^***^P ≤ 0.001 by Welch’s Student t-test. Scale bars, 50 μm.

Notably, TuJ1^+^ networks in the *Nos1*^*−/−*^ distal colon demonstrated a higher degree of branching when compared to C57BL/6J control tissues (Fig. 5a–d; Supplementary Movies 1 and 2). Importantly, the mean number of junctions observed in C57BL/6J distal colonic ENS tissue was 566.9 ± 68.5 which was comprised of 458.6 ± 52.8 triple and 71.8 ± 10.0 quadruple point junctions. By contrast, in the *Nos1*^*−/−*^ ENS 835.6 ± 52.4 (P = 0.003) junctions were observed in the distal colon which comprised 676.8 ± 40.0 triple (P = 0.002) and 104.8 ± 7.5 quadruple point junctions (P = 0.01; 30 z-projected hpf, n = 6; Fig. 5d). However, the ratio of triple to quadruple junctions in either model remained similar at ~ 6:1. Furthermore, the total number of branches was significantly greater (P = 0.004; 30 z-projected hpf, n = 6; Fig. 5e,f) in the *Nos1*^*−/−*^ distal colonic tissue (1533.4 ± 92.1) compared to control C57BL/6J mice (1069.0 ± 123.1). Additionally, the total length of branches in the *Nos1*^*−/−*^ distal colonic ENS was found to be significantly greater (23,451.2 ± 1139.5 pxls vs 15,731.4 ± 1467.8 pxls; P = 0.0001, 30 z-projected hpf, n = 6; Fig. 5g) than that of control C57BL/6J mice. However, despite differences in junction number, total branches and total branch length, mean branch length was not found to be statistically different between the C57BL/6J (24.8 ± 1.2 pxls; 30 z-projected hpf, n = 6) and *Nos1*^*−/−*^ colon (21.5 ± 1.4 pxls; 30 z-projected hpf, n = 6; p = 0.08; Fig. 5h). Together, these data suggest that global loss of nNOS results in the development of a ENS with greater interconnectivity and increased neuronal projections. Similarly, we again sought to confirm the validity of these branching analyses in the *Ednrb*^*−/−*^ colon: where progressive loss of myenteric ganglia is known to occur, from ganglionic to aganglionic regions, alongside overgrowth of extrinsic neuronal fibres. Importantly, in this well characterised model, our digital analysis was able to positively identify and quantify this graded loss of the ENS. Here, in the ganglionic region, digital analysis revealed 503 junctions (400 triple point and 76 quadruple point junctions) in ganglionic bowel. By comparison, the transitional zone (379 total junctions; 314 triple junctions, 41 quadruple junctions) and aganglionic (83 total junctions; 70 triple junctions, 8 quadruple junctions) regions displayed reduced neuronal branching (Supplementary Figure 1). Moreover, overall branch numbers were found to be reduced in a graded fashion in the *Ednrb*^*−/−*^ transitional zone (713 branches) and aganglionic (181 branches) segments compared to more proximal ganglionated gut (945 branches; Supplementary Figure 1) further supporting our digital analytical approach.

### Loss of *Nos1* has no effect on network directionality or coherency

**Figure 6:**
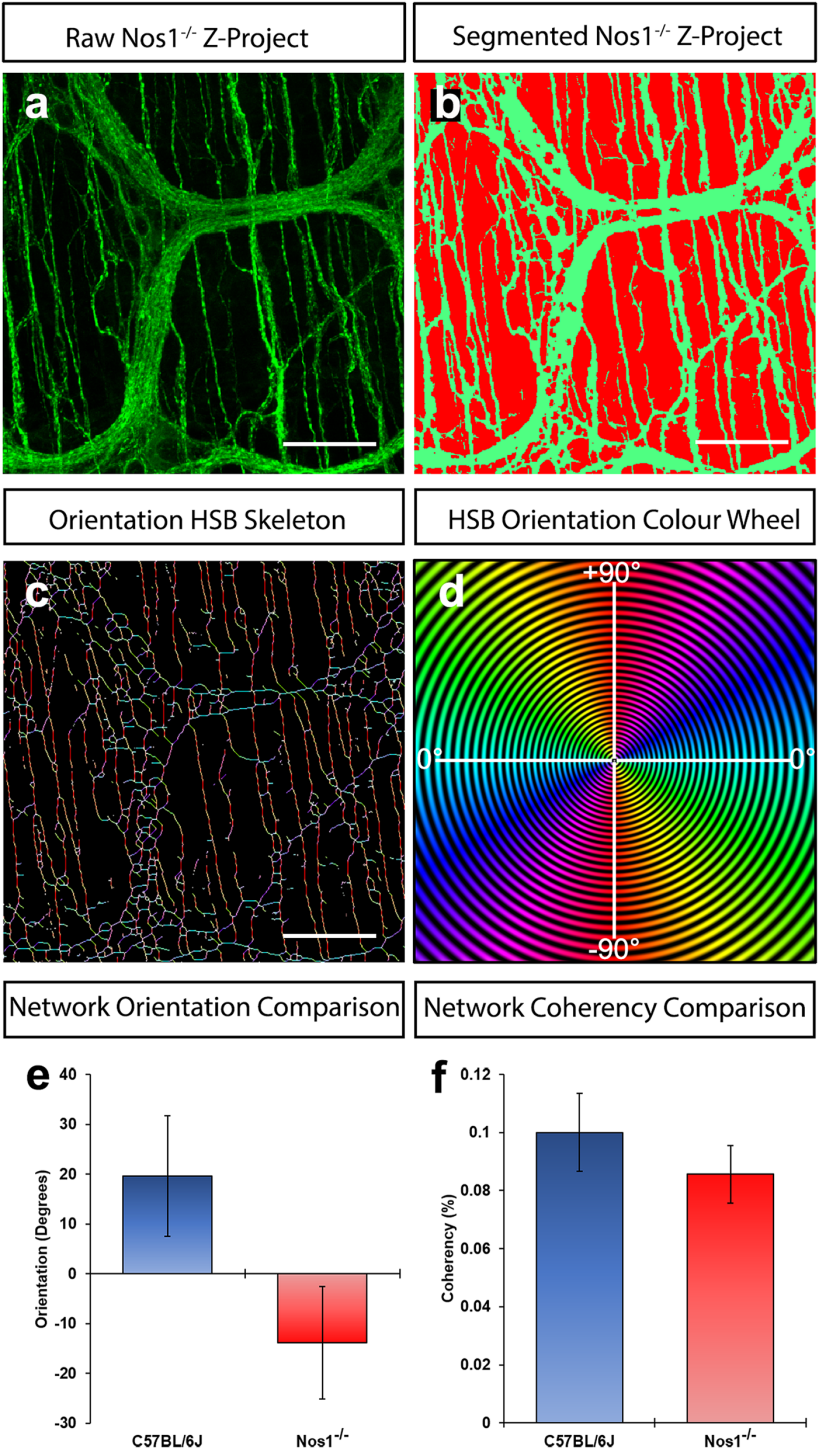
Analysis of TuJ1 network orientation and coherency. (**a**) Representative confocal z-project demonstrating the TuJ1^+^ (green) network in the *Nos1*^*−/−*^ distal colon. (**b**) Representative image of digitally segmented image in *a* showing pseudocoloured TuJ1^+^ neural network (green) and “interganglionic” regions (i.e. background; red). (**c**,**d**) Representative image (*c*) showing hue, saturation and brightness skeletonised map of network from *b* psuedocoloured based on 180° hue map scaling (*d*). Hue indicates orientation in degrees (from − 90° to + 90°), saturation indicates coherency and brightness is equivalent to the source image. (**e**) Summary data showing mean orientation of enteric fibres in C57BL/6J (blue bars) and *Nos1*^*−/−*^ (red bars) distal colon. P > 0.05 by Welch’s Student t-test. (**f**) Summary data showing coherency of enteric fibres in C57BL/6J (blue bars) and *Nos1*^*−/−*^ (red bars) distal colon. P > 0.05 by Welch’s Student t-test. Scale bars, 50 μm.

Having demonstrated that network coverage, branching and junctioning are altered in the *Nos1*^*−/−*^ distal colon we next sought to assess if these changes led to alterations in network directionality (Fig. 6a–d) and coherency (i.e., the degree to which local features are orientated)^21^. Interestingly, neither network orientation (C57BL/6J: 19.6 ± 12.1° vs *Nos1*^*−/−*^: − 13.9 ± 11.2°; P = 0.05, 30 z-projected hpf, n = 6) nor coherency (C57BL/6J: 0.10 ± 0.01% vs *Nos1*^*−/−*^: − 0.09 ± 0.01%; P = 0.40, 30 z-projected hpf, n = 6;) were found to be different (Fig. 6e,f). Although, it appeared that network directionality was different in C57BL/6J vs *Nos1*^*−/−*^ mice, this was due to an analytical artefact in which ‘up and down directionality’ were registered as ‘ ± 90°’, respectively, in relation to a fixed point. Taken together these data suggest that loss of *Nos1* has no discernible effect on the directionality of the ENS which retains ‘random’ coverage (i.e., < 1% coherency).

## Discussion

Understanding of the overall effects of neuronal loss in the ENS remains rudimentary. Often, in diagnosing gut motility dysfunction, damage to the ENS; or loss of neuronal subtypes, is attributed as the causative factor based on histopathological screening for a panel of common neural, glial and interstitial cell markers. While current methodology may reveal the apparent cause of disease, this approach offers little in network characterisation in terms of structure; barring detectable evidence of aganglionosis, hyper/hypoganglionosis or the presence of ectopic ganglia, and often ignores subtle compensatory factors with could be phenotypically relevant. Similarly, subtle compensatory mechanisms and/or occult alterations in network structure are often not examined in animal models of human disease. In both human and animal studies this is in part due to the sheer scale and complexity of the ENS. However, in not assessing if such changes occur, this reductionist approach may limit our understanding of developmental, and disease, processes within the gut. More recently, efforts have been made to bridge this gap using network quantification methods to highlight differences in neuronal density^22,23^, neuronal fibre numbers and branching using TuJ1^24–26^, or using artificial intelligence (AI)-driven tools in terms of unbiased quantification of neuronal numbers^27^. Here, we extend this AI-driven approach using machine learning methodologies to highlight that occult changes in network structure occur within the *Nos1*^*−/−*^ mouse colon. Notably, we show that, despite gross visual similarities; the *Nos1*^*−/−*^ ENS displayed morphological differences with increases in neuronal density, projections and branching when compared to age matched control mice. We also demonstrate that Vasoactive intestinal peptide (*Vip)* is upregulated*,* at the transcriptional level, upon global loss of *Nos1*; pointing to compensatory mechanisms within ENS development. Previous studies have shown that VIP displays significant colocalization within nNOS^+^ neurons in the mature ENS^28–31^. Moreover, modulation of nNOS and VIP expression in the ENS (i.e., counterbalance of increasing VIP expression upon reduction of nNOS expression) has been noted in disease states such as Parkinson’s disease^32^. Similarly, increases in VIP expression within nNOS^+^ neurons has been shown in response to both pharmacological treatment and culture^33,34^. Our findings add further weight to the hypothesis that such compensation may be a protective mechanism whereby the ENS attempts to rebalance excitatory and inhibitory pathways upon the loss of major nitrergic relaxatory signalling. However, our findings of changes in network structure also suggest that this compensatory mechanism results in significant morphological remodelling within the ENS. Taken together, our findings highlight that, even within a relatively simple transgenic animal model, major changes in ENS network structure and composition occur which can be overlooked. Extrapolating these findings to human disease states, we propose that current methodologies are insufficient to determine the true scope of phenotypically relevant changes which occur upon disruption of the ENS. We acknowledge that a major limitation of our study is the use the *Nos1*^*tm1Plh*^/J (*Nos1*^*−/−*^) knockout model for such characterisation studies. While attractive as a model, we recognise that global loss of nNOS signalling is unlikely to truly reflect disease states in the ENS; wherein loss or disruption of specific, or multiple, neuronal subtypes is likely to occur in a regionalised or sporadic manner. Nevertheless, we demonstrate that occult changes in network structure occur upon global loss of *Nos1*, however*,* the extent to which these structural changes influence physiological output is unclear. Furthermore, it is unclear if similar alterations would be observed in human disease states where additional influences such as inflammation are likely to have profound effects on ENS structure and dynamics^35–38^. Previous human studies have shown dual loss of nNOS neurons and interstitial cells of Cajal (ICC) in multiple disease states^39–42^. In keeping with these findings, our previous work has demonstrated reduction of ICC networks within the *Nos1*^*−/−*^ global knockout^20^; suggesting that loss of nNOS signalling has significant effects including remodelling of the neuromusculature. Our current findings add to these data, though raise questions as to the “cause vs consequence” of many of the remodelling effects observed in diseased tissues and animal models. Importantly, global knockout models fail to address the impact of conditional loss of nNOS, in the ENS, which is characteristic of many enteric neuropathies. Therefore, it will be important to investigate how conditional loss of neuronal subtypes impacts upon network structure and the surrounding microenvironment in this context. While the imaging protocol utilised within the current study attempted to limit sampling variation and improve consistency, we appreciate that analysis of the ENS at a larger scale would be more advantageous. Unfortunately, current technical limitations such as low spatial resolution in ‘z’, at low magnification, and vignetting in stitched high-power images, limited our ability to apply similar analytics to the ENS on a whole organ scale. Indeed, our failure to detect potential differences in network directionality is likely due to limitations in our imaging protocol, which limited the scope of any particular field of view, despite the ENS displaying well established directionality along the longitudinal axis^43,44^. Future improvements in imaging and acquisition will, however, provide further opportunities to utilise AI-driven approaches to reveal yet more detail in terms of network structure and pattern recognition in both development and disease settings.

We conclude that this study provides critical evidence of occult changes in the ENS, including structural and molecular remodelling, upon loss of nNOS signalling. Further, we propose that novel machine learning approaches, and automated computational image analysis, are beneficial in revealing possible hitherto undetected changes in ENS structures which could be phenotypically relevant.

## Methods

### Animals

6–8 week-old C57BL/6J (Stock No:000664) and B6.129S4-*Nos1*^*tm1Plh*^/J (herein *Nos1*^*−/−*^; Stock No:002986) mice were obtained from The Jackson Laboratory (Bar Harbor, USA). All animals were housed, and experiments performed, in accordance with relevant ARRIVE guidelines, the UK Animals (Scientific Procedures) Act 1986 and approved by the University College London Biological Services Ethical Review Process. Animal husbandry at UCL Biological Services was in accordance with the UK Home Office Certificate of Designation.

### Histochemistry

Following cervical dislocation, colonic tissues were removed to sterile phosphate-buffered saline (PBS, 0.01 mol L^−1^, pH 7.2 at 4 °C) and the mucosa was removed via fine dissection. Tissues were fixed in paraformaldehyde (4% w/v in 0.1 mol L^−1^ PBS) for 45 min at room temperature (RT). After fixation, tissues were washed thoroughly for 1 h in PBS (0.01 mol L^−1^, pH 7.2 at RT). NADPH Diaphorase activity was detected by incubating tissues in 0.1 mol L^−1^ PBS containing 0.05% Triton X-100, 1 mg/ml β-NADPH (Sigma, UK) and 0.5 mg/ml nitrobluetetrazolium (Sigma, UK) for 20 min at 37 °C. After staining, the tissues were washed thoroughly in PBS (0.1 mol L^−1^), mounted and examined using an Axioplan2 microscope (Zeiss, Germany).

### Immunohistochemistry

For immunohistochemistry, colonic preparations were prepared, fixed, and washed as above. Tissues were subsequently blocked for 1 h (0.1 mol L^−1^ PBS containing 1% Triton X-100, 1% Bovine Serum Albumin). Tissues were incubated in primary antibody (diluted in 0.1 mol L^−1^ PBS containing 1% Triton X-100, 1% Bovine Serum Albumin, Supplementary Table 1) for 16 h at 4 °C and immunoreactivity was detected using the secondary antibodies listed in Supplementary Table 2 (1:500 in 0.1 mol L^−1^ PBS, 1 h at RT). Before mounting, tissues were washed thoroughly in PBS (0.1 mol L^−1^ PBS for 2 h at RT). Control tissues were prepared by omitting primary or secondary antibodies. Tissues were examined using as LSM710 confocal microscope (Zeiss, Germany). Confocal micrographs were digital composites of Z-series scans or individual optical sections. Final images were constructed using FIJI software^45^.

### HuC/D^+^ neuronal quantification

HuC/D positive neuronal cell numbers were quantified, in a blinded fashion, using the “Analyze Particles” function in FIJI software^45^. Briefly, following auto-thresholding, five independent images (1 μm optical section; 512 × 512 pixels) of HuC/D^+^ cells, at the level of the myenteric plexus, were analysed in each mouse distal colon in an automated fashion using the following settings: *Size (μm*^*2*^*) 10-Infinity; Circularity 0.00–1.00*. After unblinding, average neuronal cell counts were calculated, per individual mouse, to allow inter- and intra-group comparisons.

### Neuronal network image acquisition

To allow quantitative analysis of enteric neuronal networks, five Z-series image stacks (21 × 1 μm optical sections; 512 × 512 pixels) were obtained from the distal colon in a cross-shaped array using a 40× (NA1.2) water immersion objective (Zeiss, Germany). Initially, a Z-series image stack was acquired centrally, approximately 2 cm from the internal anal sphincter, followed by four subsequent Z-series stacks obtained at a distance of 1000 μm in the proximal, distal and both lateral directions using automated directional functions in Zen software (Zeiss, Germany). Confocal micrographs were digital composites of Z-series scans. Final images were constructed using FIJI software^45^.

### Neuronal network analysis

Each Z-series (21 slices) underwent a pre-filtering process and conversion to a single frame Z-project. Five such Z-projects were produced for six mice of each cohort (i.e., 30 z-projected hpf). For each mouse, z-projects were stacked and segmented using ImageJ’s (version 1.53c) ‘Trainable Weka Segmentation’ and a classifier that was previously trained on similar images. The resulting segmentation was thresholded and/or skeletonised before standard analysis operations within ImageJ were applied, and the results exported to Excel for analysis. Analysis metrics, based on key, previously reported, characteristics of neuronal networks were assessed, including TuJ1 density, interganglionic area, and orientation. Further, to enable robust branch and junction analyses, 3D Ridge Detection was applied to produce a 3D skeletonised network which allowed inclusion of Z-plane branching in subsequent analysis. A detailed description of the processes applied can be found in Supplementary Information.

### qRT-PCR

RNA was extracted from C57BL/6J and *Nos1*^*−/−*^ tissues using TRIzol reagent (ThermoFisher, UK) and treated with DNase I (Qiagen, UK). First-strand cDNA was amplified from 1 μg RNA using SuperScript VILO cDNA Synthesis Kit (ThermoFisher, UK). qRT-PCR was performed with an ABI Prism 7500 sequence detection system (Applied Biosystems, UK) using the Quantitect SYBR Green PCR kit (Qiagen, UK), according to the manufacturer's instructions. qRT-PCR was performed in triplicate, using region-specific primers designed against mouse sequences for *Gapdh, Chat, Nos1, Sub P and Vip* (Supplementary Table 3). Gene expression data were expressed as a proportion of *Gapdh*, as a reference, using ΔΔCT calculations.

### Statistical analysis

Data are expressed as mean ± standard error of the mean. Statistical analysis was performed using GraphPad Prism software (GraphPad, USA). For quantitative real time PCR (qRT-PCR) analyses fold-change comparison between control and *Nos1*^*−/−*^ samples was performed using ΔΔCT and statistical comparison was determined using ΔCT values by Welch's t-test values. Results were considered significant at P < 0.05. The ‘n values’ reported refer to the number of colonic segments examined, each from a separate mouse.

## Supplementary Information

Supplementary Information 1.

Supplementary Video S1.

Supplementary Video S2.

## Data Availability

All authors had access to the study data and had reviewed and approved the final manuscript. The data that support the findings of this study are available from the corresponding author upon reasonable request.

## Abbreviations

AI: Artificial intelligence
Chat: Choline acetyltransferase
ENS: Enteric nervous system
GAPDH: Glyceraldehyde 3-phosphate dehydrogenase
Hpf: High power field
nNOS: Neuronal nitric oxide synthase
NO: Nitric oxide
PBS: Phosphate-buffered saline
Pxls: Pixels
qRT-PCR: Quantitative real-time polymerase chain reaction
ROI: Region of interest
RT: Room temperature
RT-PCR: Real-time polymerase chain reaction
Sub P: Substance P
Vip: Vasoactive intestinal peptide
TuJ1: Neuron-specific class III beta-tubulin
3D: 3-Dimensional
