# Supplementary material for: Automated computational analysis reveals structural changes in the enteric nervous system of nNOS deficient mice
Source: Sci Rep. 2021 Aug 25;11:17189. doi: 10.1038/s41598-021-96677-x (PMC8387485; doi:10.1038/s41598-021-96677-x)
Supplement: Supplementary file 1 — Supplementary Information 1. [file 41598_2021_96677_MOESM1_ESM.docx]

**
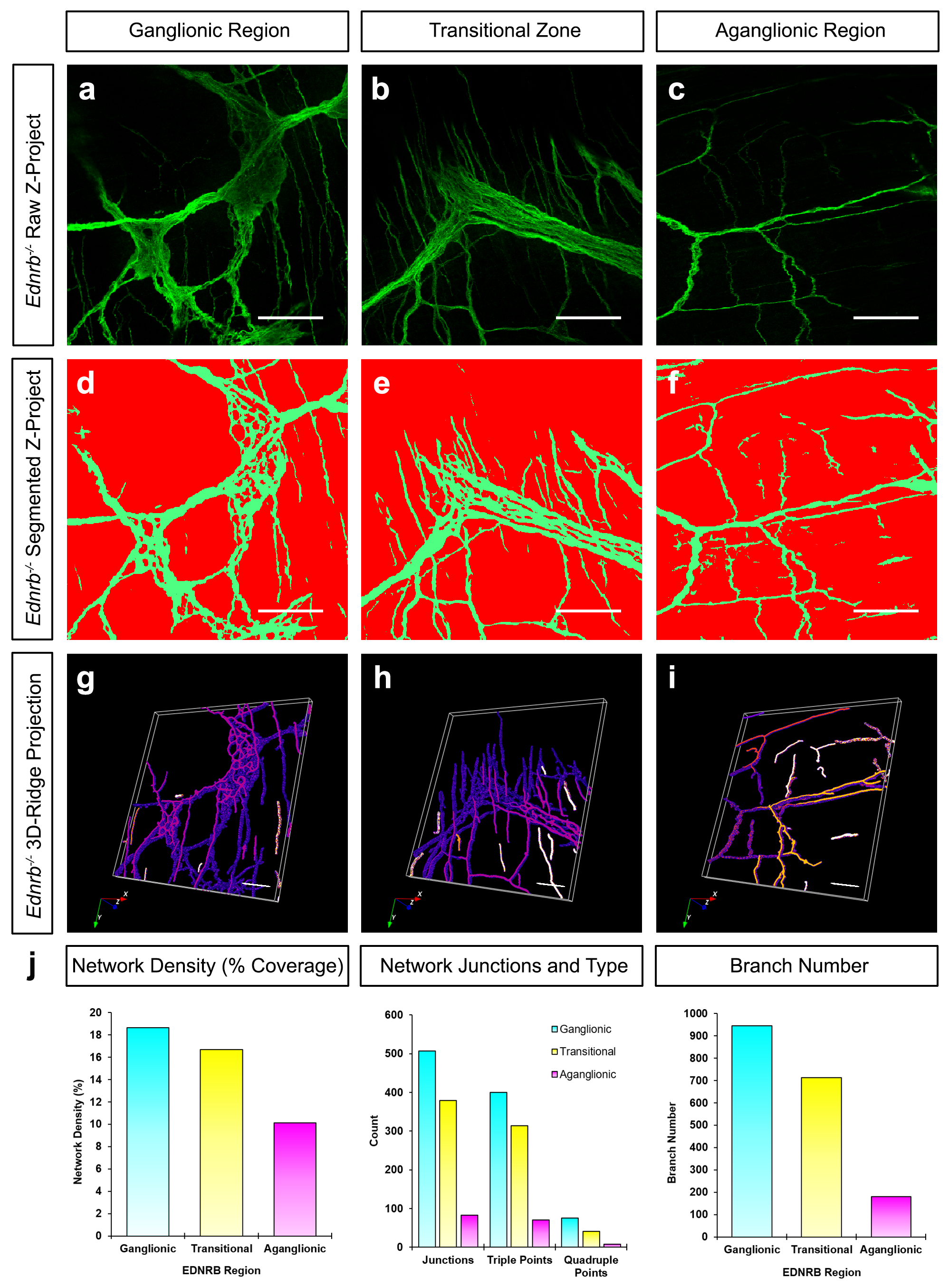
**

**Supplementary Figure 1. Validation of machine learning analysis in *Ednrb^-/-^* colon. (a-c)** Representative confocal z-projected images demonstrating the TuJ1^+^ (green) network in the *Ednrb^-/-^* (*Endrb^tm1Ywa^/*J; Jax 003295; Jackson Laboratory, USA) ganglionic (*a*), transitional zone (*b*) and aganglionic regions (*c*). **(d-f)** Representative images, of digitally segmented images in *a-c,* showing pseudocoloured TuJ1^+^ neural network (green) and “interganglionic” regions (i.e., background; red). **(g-i)** Twice eroded skeletonised versions of *d-f,* produced through ridge detection, projected in 3D and pseudocoloured in ‘mpl-plasma’. **(j)** Summary data showing network density (*left),* network junction number and type (*center*) and branch number (*right*) in *Ednrb^-/-^* ganglionic (*cyan bars*), transitional zone (*yellow bars*) and aganglionic regions (*magenta bars*). Scale bars, 50μm.

**Supplementary Movie 1.** Representative movie showing a C57BL/6J TuJ1^+^ network (green), projected in 3D, with a superimposed skeleton (magenta) produced through ridge detection.

**Supplementary Movie 2.** Representative movie showing a *Nos1^-/-^* TuJ1^+^ network (green), projected in 3D, with a superimposed skeleton (magenta) produced through ridge detection.

**Supplementary Table 1. Primary Antibodies used for Immunohistochemistry**

| **Primary Antibody** | **Concentration** | **Company** |
| --- | --- | --- |
| Mouse anti-TuJ1 | 1:500 | Covance |
| Mouse anti-HuC/D | 1:500 | Invitrogen |
| Rabbit anti-nNOS | 1:400 | Invitrogen |

**Supplementary Table 2. Secondary Antibodies used for Immunohistochemistry**

| **Secondary Antibody** | **Alexa Fluor** | **Concentration** | **Company** |
| --- | --- | --- | --- |
| Goat anti-mouse | 488 | 1:500 | Invitrogen |
| Goat anti-mouse | 568 | 1:500 | Invitrogen |
| Goat anti-rabbit | 488 | 1:500 | Invitrogen |
| Goat anti-rabbit | 568 | 1:500 | Invitrogen |
| DAPI | - | 1:1000 | Sigma |

**Supplementary Table 3. Primer Sequences used for RT-PCR**

| **Gene** | **Forward primer** | **Reverse primer** |
| --- | --- | --- |
| *Gapdh* | TCCTGCACCACCAACTGCTT | CACGCCACAGCTTTCCAGAG |
| *Nos1* | ATCTGTCTCGCCAGCCATCAGCCA | GGAGCTTTGTGCAGTTTGCCGTCG |
| *Vip* | AGGAGCAGGTGACCCTGACC | TCGCTGGTGAAAACTCCATCA |
| *Chat* | GATGAACGCCTGCCTCCAAT | GGCATACCAGGCAGATGCAG |
| *Sub P* | CCGACTGGTCCGACAGTGAC | CGCTTGCCCATTAATCCAAA |

**ENS Network Image processing and analysis scripts**

This workflow details the scripted macros used to perform digital analysis of TuJ1^+^ networks using ImageJ/FIJI including: (1) *Preprocessing*, (2) *WEKA Trainable Segmentation*, (3) *WEKA Segmentation Saving*, (4) *Network Analysis*, *(*5) *Branching and Junction Analysis* and (6) *Orientation Analysis*. These macros where scripted in the language “.ijm”. The six scripts are detailed below:

***1) Preprocessing***

This process allows for selection of specific TuJ1 channel; filtering to remove debris; conversion to a single frame and colour normalisation, to improve segmentation accuracy.

///Open/save locations files//

dir1 = getDirectory("Choose Raw Image Source Folder ");

dir2 = getDirectory("Where would you like to save the pre-processed images? ");

list = getFileList(dir1);

setBatchMode(true);

for (i=0; i<list.length; i++) {

showProgress(i+1, list.length);

open(dir1+list[i]);

//Filter images//

run("Duplicate...", "duplicate channels=2");

//setOption("ScaleConversions", true);

run("8-bit");

run("Remove Outliers...", "radius=1 threshold=5 which=Bright stack");

run("Minimum...", "radius=1 stack");

run("Gaussian Blur...", "sigma=1 stack");

run("Subtract Background...", "rolling=20 stack");

run("Z Project...", "projection=[Max Intensity]");

run("Enhance Local Contrast (CLAHE)", "blocksize=200 histogram=256 maximum=2 mask=*None*");

run("Minimum...", "radius=1");

run("Color Balance...");

run("Enhance Contrast", "saturated=0.35");

run("Apply LUT");

saveAs("TIFF", dir2+list[i]);

close();

}

***2) WEKA Trainable Segmentation***

This workflow allows generation or application of a premade classifier to the preprocessed image stack in order to segment the network from the background in a semi-automated fashion.

*Note: These images will not be automatically saved. Please see macro “**3”** for this functionality.

//Open images in directory//

dir = getDirectory( "Choose the Image Folder to be Segmented" );

list = getFileList( dir );

for ( i=0; i<list.length; i++ ) {

open( dir + list[i] );

}

//Convert to stack//

run("Images to Stack", "name=Stack title=[] use");

//Duplicates stack to overcome WEKA GUI closing bug which prevents renaming.//

run("Duplicate...", "title=Stack duplicate");

//Open WEKA//

run("Trainable Weka Segmentation");

//Wait for segmentor to load in before applying settings//

wait(3000)

selectWindow("Trainable Weka Segmentation v3.2.34");

call("trainableSegmentation.Weka_Segmentation.setFeature", "Bilateral=true");

call("trainableSegmentation.Weka_Segmentation.setFeature", "Kuwahara=true");

call("trainableSegmentation.Weka_Segmentation.setMembraneThickness", "3");

call("trainableSegmentation.Weka_Segmentation.changeClassName", "0", "Background");

call("trainableSegmentation.Weka_Segmentation.changeClassName", "1", "TUJ1");

call("trainableSegmentation.Weka_Segmentation.setClassBalance", "true");

waitForUser("Train the classifier before continuing. Using the tools, select regions and add to corresponding class.")

//Create a segmented result//

call("trainableSegmentation.Weka_Segmentation.getResult");

selectWindow("Trainable Weka Segmentation v3.2.34");

close();

//Transfer labels from original stack to new segmented images//

//The code for function was adapted from code produced by Christophe Leterrier, which is available at https://imagej.nih.gov/ij/macros/misc/Transfer_Labels.txt //

{

nDEST=nSlices;

if (nDEST==1) exit("Destination is not a stack");

DEST=getTitle;

setBatchMode(true);

if (nImages<1) exit("Not enough images");

TITLES=newArray(nImages);

for (i=1; i<=nImages; i++) {

selectImage(i);

TITLES[i-1]=getTitle;

}

selectImage("Stack");

nStack=nSlices;

if (nStack!=nDEST) exit("Source and destination do not have the same slice number !");

LABELS=newArray(nStack);

for (i=0; i<nStack; i++) {

setSlice(i+1);

LAB=getInfo("slice.label");

LAB_LENGTH=lengthOf(LAB);

LABELS[i]=substring(LAB, 0,LAB_LENGTH);

}

selectImage(DEST);

for (i=0; i<nStack; i++) {

setSlice(i+1);

setMetadata("Label", LABELS[i]);

}

setBatchMode("exit and display");

}

setBatchMode(true);

{selectWindow("Stack");

run("Close");

}

setBatchMode(false);

***3) WEKA Segmentation Saving***

This procedure allows saving of the newly segmented images to a user designated folder, after renaming.

*Note: This is included as a separate and additional script to macro “**2**” due to a ‘hanging’ bug in ImageJ.

//Close the original duplicated stack//

setBatchMode(true);

{selectWindow("Stack");

run("Close");

}

setBatchMode(false);

//Separate the Classified image stack (n=5) into its constituent images//

selectWindow("Classified image");

run("Stack to Images");

// Choose the save location and get image IDs of all open images//

dir = getDirectory("Where do you want to save the segmented images?");

ids=newArray(nImages);

for (i=0;i<nImages;i++) {

selectImage(i+1);

title = getTitle;

print(title);

ids[i]=getImageID;

//Save images//

saveAs("tiff", dir+title);

}

run("Close All");

beep()

}

***4) Network Analysis***

This series of scripts allows for thresholding of inputted images after segmentation, with output data relating to *Network Coverage and* *Interganglionic Area.* This data can be subsequently exported to Excel for analysis. The “ResultsToExcel" (https://imagej.net/User:ResultsToExcel) ImageJ plugin is required for this function.

//Network Coverage//

//Open images in directory//

dir1 = getDirectory("What Folder Do You Want to Analyse? ");

list = getFileList(dir1);

setBatchMode(true);

for (i=0; i<list.length; i++) {

showProgress(i+1, list.length);

open(dir1+list[i]);

//Threshold Network Coverage//

run("8-bit");

setAutoThreshold("Default dark");

run("Threshold...");

setThreshold(121, 255);

setOption("BlackBackground", true);

run("Convert to Mask", true);

//Measure//

run("Set Measurements...", "area area_fraction limit add redirect=None decimal=3");

run("Measure");

//Saving Data to Excel//

run("Read and Write Excel", "sheet=Area");

//Close active image//

close();

//Close results window//

requires("1.30e");

if (isOpen("Results"))

selectWindow("Results");

run("Close" );

}

setBatchMode(false);

selectWindow("Threshold");

run("Close" );

///Interganglionic Area//

dir2 = getDirectory("Where would you like to save the ROI interganglionic area images? ");

list = getFileList(dir1);

setBatchMode(true);

for (i=0; i<list.length; i++) {

showProgress(i+1, list.length);

open(dir1+list[i]);

//Threshold Interganglionic Areas//

run("8-bit");

setAutoThreshold("Default dark");

run("Threshold...");

setThreshold(85, 154);

setOption("BlackBackground", true);

run("Convert to Mask");

//Analyse Interganglionic Areas//

run("Analyze Particles...", "size=20-Infinity pixel display clear add");

//Export results to Excel//

run("Read and Write Excel", "sheet=Interganglionic Area");

//Generate ROI image//

run("Flatten");

saveAs("TIFF", dir2+list[i]);

close();

//Close results window//

requires("1.30e");

if (isOpen("Results"))

selectWindow("Results");

run("Close" );

}

setBatchMode(false);

close();

//Close threshold window//

requires("1.30e");

if (isOpen("Threshold"))

selectWindow("Threshold");

run("Close" );

}

selectWindow("Threshold");

***5) Branching and Junction Analysis***

This workflow allows generation of a 3D network, via ridge detection; skeletonization of the 3D ridge network and subsequent ‘skeleton analysis’. This data can be subsequently exported to Excel for analysis. The “ResultsToExcel" (<https://imagej.net/User:ResultsToExcel>) and Neuroanatomy (<https://imagej.net/Neuroanatomy>) ImageJ plugins are required for this function.

// "Network Branching Analysis"//

dir1 = getDirectory("Choose Raw Image Source Folder ");

dir2 = getDirectory("Where would you like to save the 3D ridge skeleton? ");

dir3 = getDirectory("Where would you like to save the 3D true (1 pxl) skeleton images? ");

list = getFileList(dir1);

setBatchMode(true);

for (i=0; i<list.length; i++) {

showProgress(i+1, list.length);

open(dir1+list[i]);

originalName = getTitle();

run("Duplicate...", "duplicate channels=2");

run("8-bit");

run("Subtract Background...", "rolling=20 stack");

run("Enhance Local Contrast (CLAHE)", "blocksize=200 histogram=256 maximum=2 mask=*None*");

run("Minimum 3D...", "x=1 y=1 z=1");

run("Enhance Contrast...", "saturated=0.2 normalize process_all use");

wait(1000);

run("Gaussian Blur 3D...", "x=1 y=1 z=1");

run("Ridge Detection", "line_width=11 high_contrast=150 low_contrast=5 extend_line make_binary method_for_overlap_resolution=NONE sigma=3 lower_threshold=0.10 upper_threshold=0.80 minimum_line_length=2 maximum=5000 stack");

rename("Ridges");

run("Duplicate...", "title=[Ridges to blur] duplicate");

run("Maximum 3D...", "x=2 y=2 z=4");

run("Duplicate...", "title=threshold duplicate");

setAutoThreshold("Huang dark stack");

setOption("BlackBackground", true);

run("Convert to Mask", "method=Huang background=Dark black");

run("Duplicate...", "title=Skel duplicate");

run("Analyze Skeleton (2D/3D)", "prune=[shortest branch] prune_0 display");

selectWindow("Skel-labeled-skeletons");

rename(originalName);

close("\\Others");

//Saving//

saveAs("TIFF", dir2+list[i]);

run("8-bit");

run("Skeletonize (2D/3D)");

close("\\Others");

//Save skeleton//

saveAs("TIFF", dir3+list[i]);

run("8-bit");

run("Summarize Skeleton");

Table.rename("Skeleton Stats", "Results");

run("Read and Write Excel", "sheet=Branches");

close("*");

setBatchMode(false);

}

beep();

***6) Orientation Analysis***

This procedure thresholds and generates a sketonised network, allocates a hue to each branch and outputs (1) a pseudocloured hue image, (2) average orientation, (3) average coherency and (4) an orientation histogram. The “OrentationJ” (http://bigwww.epfl.ch/demo/orientation/) and “ResultsToExcel" (https://imagej.net/User:ResultsToExcel) ImageJ plugins are required for this script.

//Open images in directory//

dir1 = getDirectory("Choose Segmented image folder ");

dir2 = getDirectory("Where would you like to save the directionality images? ");

list = getFileList(dir1);

setBatchMode(true);

for (i=0; i<list.length; i++) {

showProgress(i+1, list.length);

open(dir1+list[i]);

//Threshold Network Coverage//

run("8-bit");

setAutoThreshold("Default dark");

run("Threshold...");

setThreshold(121, 255);

setOption("BlackBackground", true);

run("Convert to Mask", true);

//Generate Skelton Image//

run("Skeletonize");

//Calculate dominant direction and coherency//

run("OrientationJ Dominant Direction");

//Rename data table for Excel export//

Table.rename("Results");

run("Read and Write Excel", "sheet=Directionailty");

//Generate hue image to be saved//

run("OrientationJ Analysis", "tensor=1.0 gradient=0 color-survey=on hsb=on hue=Orientation sat=Coherency bri=Original-Image radian=on ");

saveAs("TIFF", dir2+list[i]);

run("Close All");

}

{

requires("1.30e");

if (isOpen("Results")

selectWindow("Results");

run("Close" );

}

{

requires("1.30e");

if (isOpen("Threshold")

selectWindow("Threshold");

run("Close" );

}

dir2 = getDirectory("Where would you like to save the Orientation Histogram images? ");

list = getFileList(dir1);

setBatchMode(true);

for (i=0; i<list.length; i++) {

showProgress(i+1, list.length);

open(dir1+list[i]);

run("8-bit");

setAutoThreshold("Default dark");

run("Threshold...");

setThreshold(121, 255);

setOption("BlackBackground", true);

run("Convert to Mask", true);

run("Skeletonize");

//Generate histogram to be saved//

run("OrientationJ Distribution");

run("OrientationJ Distribution", "tensor=2.0 gradient=0 radian=on histogram=on min-coherency=0.0 min-energy=0.0 ");

selectWindow("OJ-Histogram-1-slice-1");

saveAs("TIFF", dir2+list[i]);

close();

run("Close All");

}

setBatchMode(false);

}
